# The interaction of S100A16 and GRP78 actives endoplasmic reticulum stress-mediated through the IRE1α/XBP1 pathway in renal tubulointerstitial fibrosis

**DOI:** 10.1038/s41419-021-04249-8

**Published:** 2021-10-13

**Authors:** Runbing Jin, Anran Zhao, Shuying Han, Dan Zhang, Hui Sun, Min Li, Dongming Su, Xiubin Liang

**Affiliations:** 1 Department of Pathophysiology, Nanjing Medical University, Nanjing, 211166 China; 2 Department of Pathology, Nanjing Medical University, Nanjing, 211166 China; 3 Department of Nephrology, The Affiliated Sir Run Run Hospital of Nanjing Medical University, Nanjing, 211166 China

**Keywords:** Stress signalling, Experimental models of disease

## Abstract

Recent studies have indicated that the development of acute and chronic kidney disease including renal fibrosis is associated with endoplasmic reticulum (ER) stress. S100 calcium-binding protein 16 (S100A16) as a novel member of the S100 family is involved in kidney disease; however, few studies have examined fibrotic kidneys for a relationship between S100A16 and ER stress. In our previous study, we identified GRP78 as a protein partner of S100A16 in HK-2 cells. Here, we confirmed a physical interaction between GRP78 and S100A16 in HK-2 cells and a markedly increased expression of GRP78 in the kidneys of unilateral ureteral occlusion mice. S100A16 overexpression in HK-2 cells by infection with Lenti-S100A16 also induced upregulation of ER stress markers, including GRP78, p-IRE1α, and XBP1s. Immunofluorescence staining demonstrated that the interaction between S100A16 and GRP78 predominantly occurred in the ER of control HK-2 cells. By contrast, HK-2 cells overexpressing S100A16 showed colocalization of S100A16 and GRP78 mainly in the cytoplasm. Pretreatment with BAPTA-AM, a calcium chelator, blunted the upregulation of renal fibrosis genes and ER stress markers induced by S100A16 overexpression in HK-2 cells and suppressed the cytoplasmic colocalization of GRP78 and S100A16. Co-immunoprecipitation studies suggested a competitive binding between S100A16 and IRE1α with GRP78 in HK-2 cells. Taken together, our findings demonstrate a significant increase in S100A16 expression in the cytoplasm following renal injury. GRP78 then moves into the cytoplasm and binds with S100A16 to promote the release of IRE1α. The subsequent phosphorylation of IRE1α then leads to XBP1 splicing that activates ER stress.

## Introduction

The main pathological features of renal fibrosis are the proliferation of renal interstitial fibroblast and the accumulation of extracellular matrix (ECM). Recent studies have shown that extracellular matrix accumulates in kidney cells in response to multiple factors, including the epithelial-mesenchymal transition (EMT) [1, 2], transforming growth factor (TGF)-β signaling [3], inflammation, oxidative stress [4], and Endoplasmic reticulum (ER) stress [5, 6]. ER stress inhibitors can significantly attenuate the occurrence and development of fibrosis [7–9].

The ER is the membrane system of the cytoplasm, connected with the cell membrane and nuclear membrane. The main function of ER is protein synthesis and lipids synthesis, folding trafficking and modification [6]. ER stress signaling, also called the unfolded protein response (UPR), is a cellular adaptive mechanism that occurs in response to the disruption of ER homeostasis. Glucose-regulated protein 78 kD (GRP78) is also known as immunoglobulin heavy chain binding protein (Bip), is an important molecular chaperone located on the ER and is a member of the heat shock protein 70 families. The molecular sequence structure of GRP78 is highly conserved in many biological species. GRP78 participates in preventing the aggregation of new peptides, regulating ER calcium homeostasis, and assisting in a wide range of folding processes through two domains, namely nucleotide-binding domain (NBD) and substrate-binding domain (SBD) [10]. During cellular homeostasis, GRP78 binds to the three ER stress sensors including protein kinase RNA-like ER kinase (PERK), inositol-requiring endoribonuclease 1α (IRE1α), and activating transcription factor 6 (ATF6). The PERK autophosphorylation activated the PERK-signaling pathway, and then phosphorylates eukaryotic initiation factor-2 α (eIF2α). In turn, p-eIF2α promotes the expression of downstream proteins [11]. Similarly, the IRE1 autophosphorylation activated IRE1 pathway, and p-IRE1 drives unconventional splicing of X-box-binding protein 1 (XBP1) mRNA [6]. For ATF6 signaling, ATF6 cleavage by Site 1 protease and Site 2 protease in the Golgi apparatus-initiated ATF6 signaling pathway. The cleaved subunit (p50ATF6) works as a transcription factor [12]. Accumulation of misfolded proteins in the ER lumen induces a range of ER dysfunctions and stress [13]. In order to prevent unfolded protein accumulation, cells suffered from ER stress will initiate the UPR. It can reduce cell damage and repair homeostasis damage by suspending protein synthesis in cells, secreting protease to degrade misfolded proteins, and adding chaperones to reset the wrong proteins to normal proteins.

We have previously reported that the UPR modulator protein GRP78 binds with S100 calcium-binding protein 16 (S100A16), a novel EF-hand Ca^2+^ binding protein of the S100 family [14]. S100A16 is widely expressed in various human tissues, where it functions to promote the proliferation and lipogenesis of preadipocytes and regulate EMT of cancer cells, and reduce glucose uptake stimulated by insulin [15, 16]. We also found that the protein expression of S100A16 is significantly increased in the kidneys of unilateral ureteral occlusion (UUO) mice, an animal model of kidney fibrosis, and that the characteristic pathological changes of renal tubulointerstitial fibrosis appeared in the kidneys of S100A16 transgenic mice, indicating a positive relationship between S100A16 and tubulointerstitial fibrosis. Moreover, S100A16 was also highly expressed in kidney biopsy specimens from patients with various forms of clinical nephropathy [14].

These findings suggested that S100A16 regulates the EMT process of renal tubular cells by promoting the reorganization of the cytoskeleton-associated with renal tubulointerstitial fibrosis [14]. However, a role for S100A16 in ER stress in the fibrotic kidney has not been established. In the present study, we demonstrate that S100A16 and GRP78 are both highly expressed in HK-2 cells following induction by TGF-β1. Overexpression of S100A16 significantly accentuates the progression of renal fibrosis by upregulating the IRE1α/XBP1 pathways in HK-2 cells.

## Results

### GRP78 is markedly upregulated in kidney of UUO mice

**Fig. 1:**
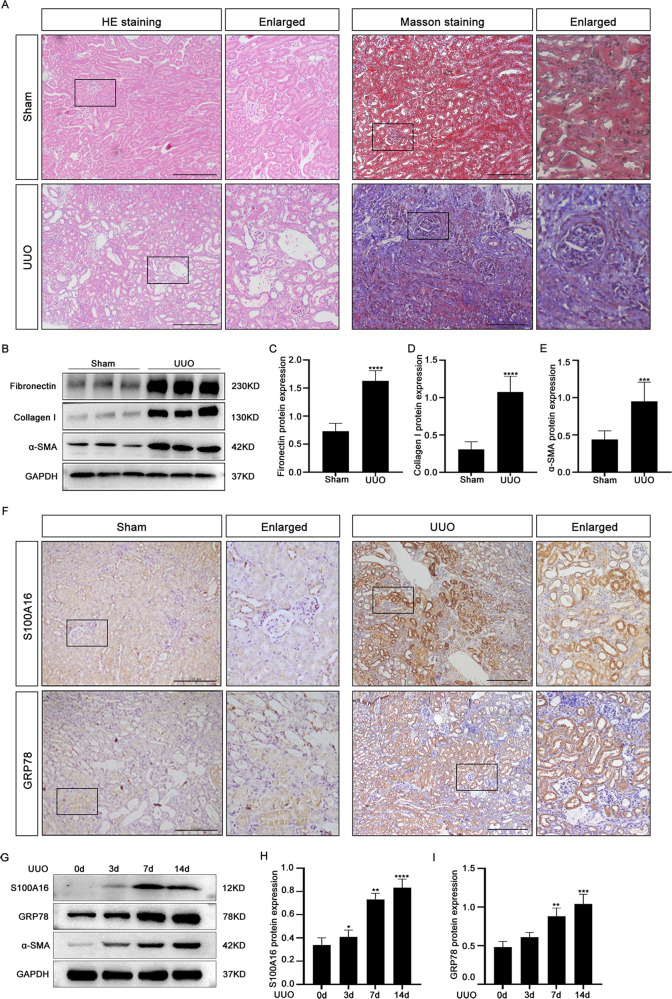
The expression of GRP78 is markedly increased in kidneys of UUO mice. **A** HE and Masson’s trichrome staining of kidney tissue in the sham operation and UUO mouse models. Scale bar = 100 μm. **B** Representative bands of western blots showing the expression of fibronectin, collagen I, and α-SMA in the kidneys of wild-type and kidney-obstructed UUO mice. **C**–**E** Quantitation of fibronectin, collagen I, and α-SMA protein expression in **B**. *p < 0.05, **p < 0.01 vs. wild-type sham groups. **F** Immunohistochemical staining of GRP78 and S100A16 in the Sham operation and UUO mice models. Scale bar = 100 μm. **G** The expressions of GRP78, S100A16, and α-SMA were determined by western blotting of proteins from UUO mouse kidneys at 3, 7, and 14 days after UUO and comparing them with proteins from a control group. **H**–**I** Quantitation of GRP78 and S100A16 expressions in **G**, normalized to GAPDH expression.

We previously demonstrated that S100A16 promotes renal tubulointerstitial fibrosis in UUO mice, and we identified Bip/GRP78 as an S100A16-interacting protein by mass spectrometry (LC-MS/MS) [14]. In this study, we first examined the expression of GRP78 in UUO mice, an admittedly animal model of interstitial fibrosis. UUO kidney sections stained with HE and Masson’s trichrome showed positive interstitial inflammation and tubulointerstitial fibrosis compared with sham kidney (Fig. 1A). There was the upregulated expression of the renal fibrosis-related genes fibronectin, collagen I, and α-smooth muscle actin (α-SMA) detected by western blotting (Fig. 1B–E).

Immunoblot data and pathologic staining confirmed the successful establishment of the UUO mouse model of renal tubulointerstitial fibrosis. Immunohistochemical (IHC) staining of kidney tissues after UUO showed clearly increased expressions of both S100A16 and GRP78 compared to sham-operated mouse kidneys (Fig. 1F). Western blots revealed a time-dependent upregulation of both S100A16 and GRP78 after UUO (Fig. 1G). The quantified data for the expression of S100A16 and GRP78 (Fig. 1H and I) agreed with the IHC data (Fig. 1F).

### S100A16 upregulation and ER stress were induced by TGF-β1 in HK-2 cells

**Fig. 2:**
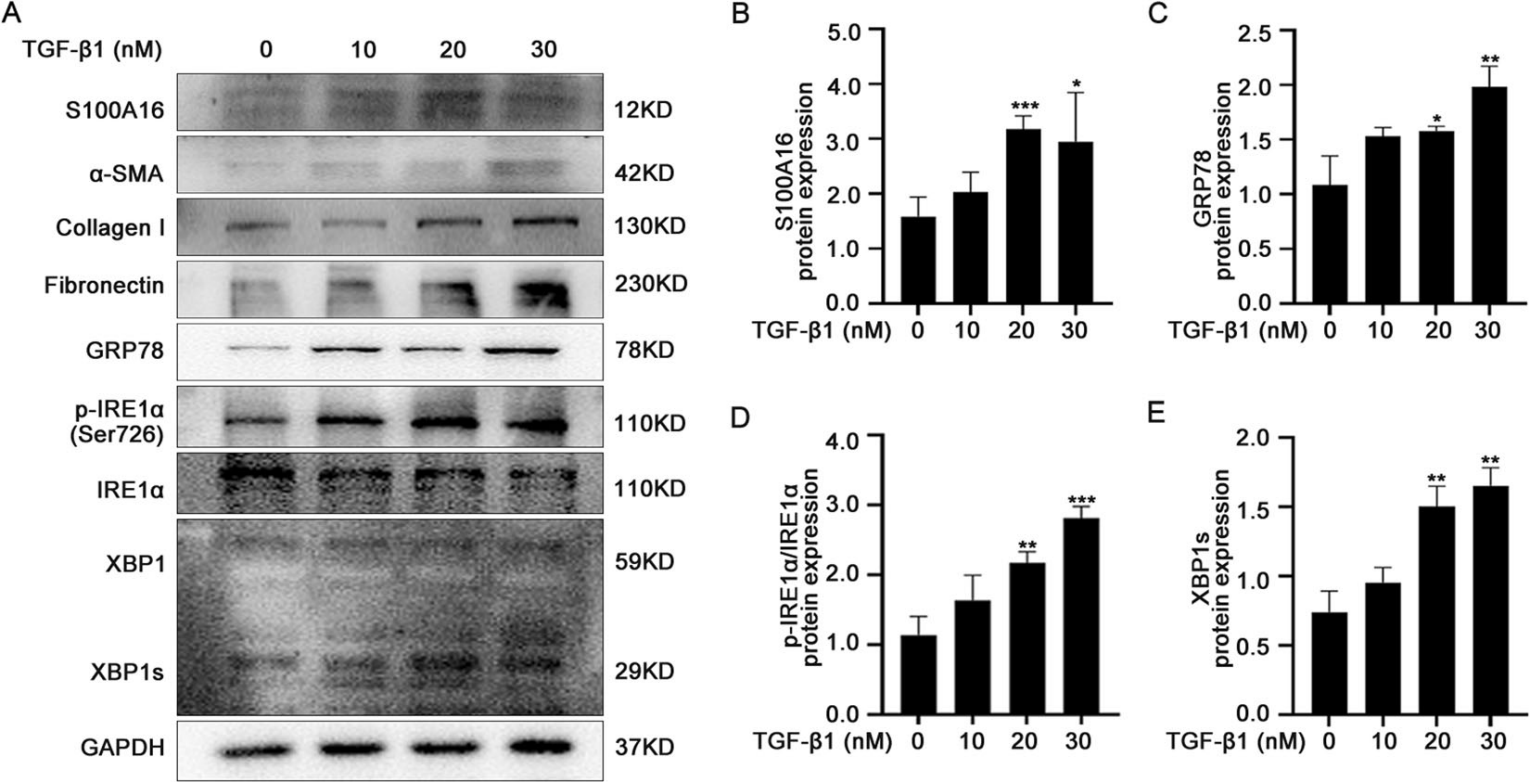
TGF-β1 induced S100A16 upregulation and ER stress in HK-2 cells. **A** TGF-β1 treatment dose-dependently increased the expressions of S100A16, fibronectin, collagen I, α-SMA, and ER stress-related proteins in HK-2 cells. **B**–**E** Quantitation of S100A16, GRP78, XBP1s, p-IRE1α/IRE1α protein expression in **A**, normalized to GAPDH expression. Bars are means ± SE from three independent experiments. *P < 0.05, **P < 0.01, ***P < 0.001.

Exposure of HK-2 cells to TGF-β1 (0, 10, 20, and 30 nM) increased the expression of S100A16 and the fibrosis-related proteins fibronectin, collagen I, and α-SMA (Fig. 2A). The ER stress markers GRP78, p-IRE1α, and XBP1s also showed markedly increased expression following TGF-β1 treatment (Fig. 2A). Quantitation of the western blot data for S100A16 and GRP78 is shown in Fig. 2B and C. Real-time PCR of HK-2 cells treated with TGF-β1 showed increased mRNA expression of GRP78 and S100A16, in agreement with the protein expression data (Fig. S1a). Quantitation of the ratio of p-IRE1α and IRE1α and XBP1s protein in Fig. 2A is shown in Fig. 2D and E.

### S100A16 physically interacts with GRP78 in HK-2 cells

**Fig. 3:**
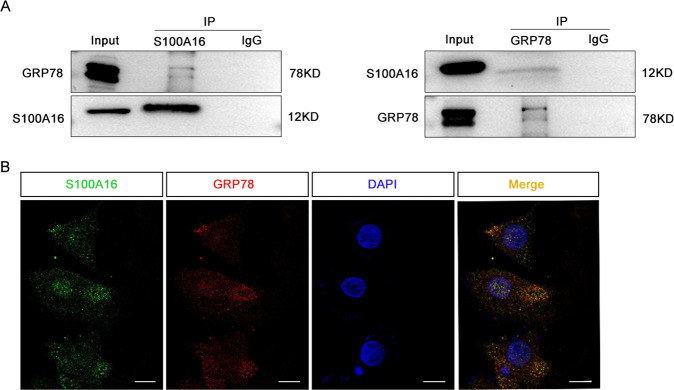
S100A16 physically interacts with GRP78 in HK-2 cells. **A** An interaction between GRP78 and S100A16 was detected by co-immunoprecipitation analysis in normal HK-2 cells. **B** The colocalization of S100A16 and GRP78 was detected in HK-2 cells by immunofluorescence staining. S100A16 in green, GRP78 in red, and DAPI in blue. Scale bar = 20 μm.

Transgenic expression of S100A16 in mice (S100A16^Tg^ mice) resulted in expression of both S100A16 and GRP78 in S100A16^Tg^ kidneys and further augmentation of S100A16 and GRP78 expression in S100A16^Tg^ mice after UUO (Fig. S1b). Co-immunoprecipitation (Co-IP) analysis performed using S100A16 antibodies to isolate the protein complex from HK-2 cells, followed by blotting using GRP78 antibodies, confirmed the interaction between GRP78 and S100A16 (Fig. 3A). The physical interaction between S100A16 and GRP78 was detectable under endogenous conditions and was further supported by IP experiments using GRP78 antibodies and blotting with S100A16 antibodies (Fig. 3A). Notably, immunofluorescence (IF) staining images indicated a marked colocalization of S100A16 and GRP78 (Fig. 3B), in agreement with the western blotting data.

### S100A16 participates in endoplasmic reticulum stress in HK-2 cells through IRE1α/XBP1 pathway

**Fig. 4:**
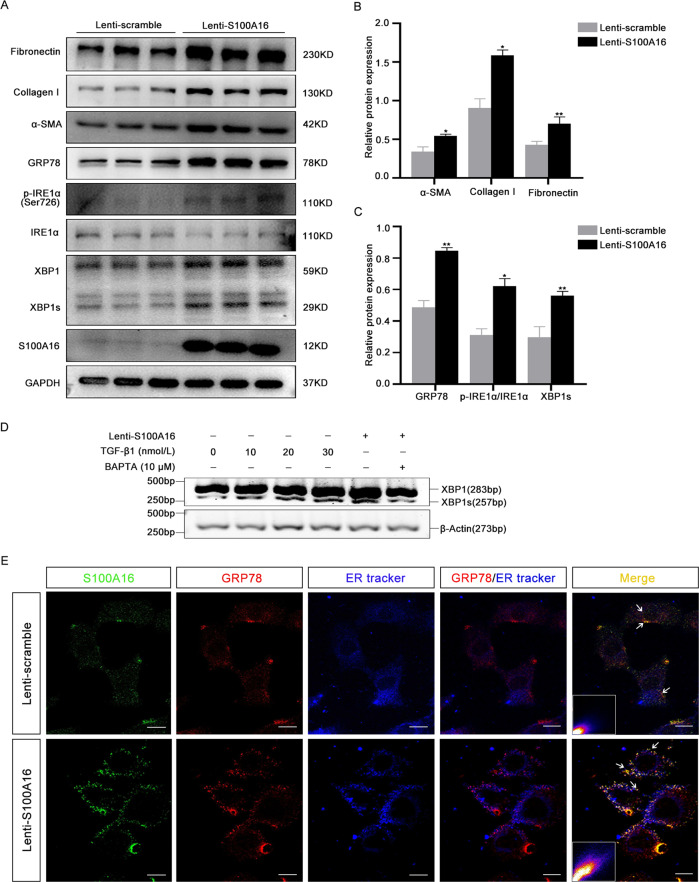
S100A16 participates in endoplasmic reticulum stress in HK-2 cells through IRE1α/XBP1 pathway. **A** Expressions of the renal fibrosis-related genes fibronectin, collagen I, and α-SMA, and the ER stress-related genes GRP78, p-IRE1α, XBP1, and XBP1s in S100A16-overexpressing and Lenti-scramble HK-2 cells. **B** Quantitation of fibronectin, collagen I, and α-SMA protein expression in **A**, normalized to GAPDH expression. **C** Quantitation of GRP78, XBP1s, and p-IRE1α/IRE1α protein expression in a, normalized to GAPDH expression. Bars indicate the means ± SE from three independent experiments. *P < 0.05, **P < 0.01, ***P < 0.001. **D** RT-PCR analysis of mRNA levels of spliced XBP1 in SA100A16-overexpressing HK-2 cells treated with TGF-β1 (0, 10, 20, and 30 nM) and/or with BAPTA-AM. **E** Immunofluorescence staining showing cellular colocalization of S100A16 and GRP78. S100A16 in green, GRP78 in red, and ER tracker in blue. Scale bar = 20 µm. The lower corner in the merged panel shows the pixels indicating colocalization of GRP78 and S100A16 in S100A16-overexpressing and Lenti-scramble HK-2 cells. The GRP78 and S100A16 colocalization pixels were identified by the Colocalization Finder plugin in ImageJ. Scale bar = 20 μm.

As shown in Fig. 4A, western blotting results showed that S100A16 overexpression in HK-2 cells dramatically promoted the upregulation of fibronectin, collagen I, and α-SMA compared with Lenti-scramble infected HK-2 cells, in agreement with our previous study [14].

GRP78, p-IRE1α, and XBP1s expressions were also markedly increased in HK-2 cells overexpressing S100A16. Quantitation of the western blot data for fibronectin, collagen I, and α-SMA is shown in Fig. 4B and for GRP78, p-IRE1α/IRE1α, and XBP1s in Fig. 4C. RT-PCR analysis of spliced XBP1 in S100A16-overexpressing HK-2 cells revealed a dose-dependent increase in XBP1 mRNA in response to TGF-β1 (0, 10, 20, and 30 nM) (Fig. 4D). The upregulation of XBP1s level by S100A16 overexpression was attenuated by the addition of BAPTA-AM, a calcium chelator. No significant changes were noted in other ER stress markers belonging to the PERK/eIF2α and ATF6 pathways, including ATF6, p-PERK, and CHOP (Fig. S1c). These findings indicated that S100A16 participates in ER stress in HK-2 cells through the IRE1α/XBP1 pathway.

IF staining of GRP78 and S100A16 in HK-2 cells revealed that the interaction between GRP78 and S100A16 predominantly occurs in the ER of control HK-2 cells (Fig. 4E). Overexpression of S100A16 by HK-2 cells resulted in colocalization of S100A16 and GRP78 mainly in the cytoplasm and at the cell membrane, rather than in the ER (Fig. 4E). These results suggested that there was a dynamic interaction between GRP78 and S100A16 in response to ER stress.

### Ca^2+^ was required for ER stress-induced by S100A16 in HK-2 cells

**Fig. 5:**
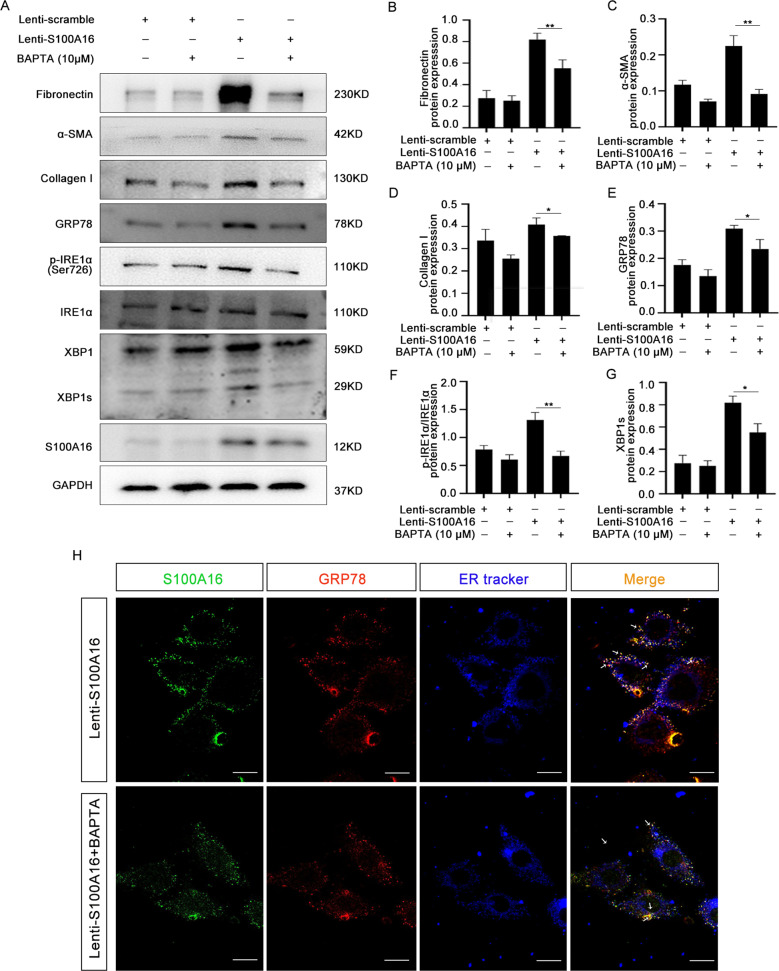
Ca^2+^ was required for ER stress-induced by S100A16 in HK-2 cells. **A** Immunoblots showing the expression of renal fibrosis and ER stress-related genes in Lenti-scramble and S100A16-overexpressing HK-2 cells pretreated with BAPTA-AM (10 μM). **B**–**G** Quantitation of fibronectin, collagen I, α-SMA, GRP78, p-IRE1α/IRE1α, and XBP1s protein expression in **A**, normalized to GAPDH expression. Bars are means ± SE from three independent experiments. *P < 0.05, **P < 0.01, ***P < 0.001. **H** The cellular colocalization of S100A16 and GRP78. S100A16 in green, GRP78 in red, and ER tracker in blue. Scale bar = 20 µm.

HK-2 cells stably overexpressing S100A16 showed an increased Ca^2+^ fluorescence signal with the Rhod-2 AM fluorescent probe, but this signal was abolished by pretreatment with 10 µM BAPTA-AM, a Ca^2+^ chelator (Fig. S2a). BAPTA-AM pretreatment also attenuated the upregulation of fibronectin, α-SMA, collagen I, and ER stress markers induced by S100A16 overexpression in HK-2 cells (Fig. 5A). Quantitation of the western blot data for fibronectin, α-SMA, collagen I, GRP78, p-IRE1α/IRE1α, and XBP1s is shown in Fig. 5B–G. The mRNA levels of GRP78 and S100A16, normalized to GAPDH, were also consistently attenuated by BAPTA-AM in HK-2 cells overexpressing S100A16 (Fig. S2b). The upregulation of XBP1s by S100A16 overexpression was blunted by the addition of BAPTA-AM (Fig. 4D). IF staining to examine the colocalization of GRP78 and S100A16 revealed that BAPTA-AM pretreatment decreased the merged signals of S100A16 and GRP78 within the ER in HK-2 cells (Fig. 5H).

### The interaction of GRP78 with S100A16 releases IRE1α in HK-2 cells

**Fig. 6:**
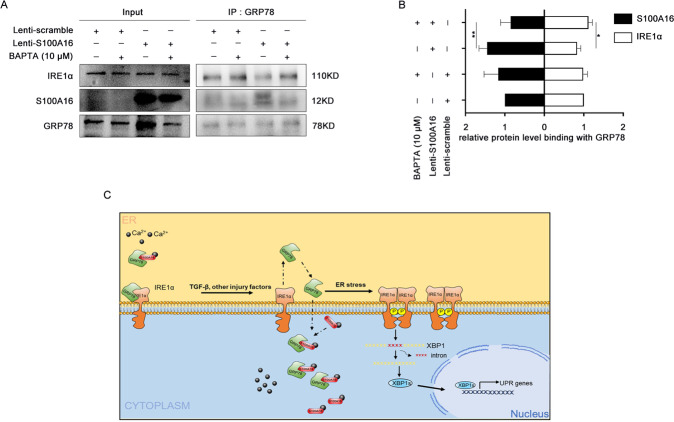
The interaction of GRP78 with S100A16 releases IRE1α in HK-2 cells. **A** Immunoprecipitation (IP) analysis demonstrating an interaction between GRP78 and IRE1α as well as GRP78 and S100A16 after treatment with BAPTA-AM (10 μM) in normal and S100A16-overexpressing HK-2 cells. **B** Quantitation of IRE1α and S100A16 protein expression in **A**, normalized to GRP78 expression. Bars are means ± SE from four independent experiments. *P < 0.05, **P < 0.01, ***P < 0.001. **C** Schematic of the interaction between S100A16 and GRP78 and activation of endoplasmic reticulum stress-mediated through the IRE1α/XBP1 pathway in renal tubulointerstitial fibrosis.

Co-IP assays using GRP78 antibodies to isolate protein complexes, followed by blotting using antibodies to IRE1α, S100A16, and GRP78, revealed a marked decrease in the binding between IRE1α and S100A16 in S100A16-overexpressing HK-2 cells, but an increased binding of IRE1α to GRP78 (Fig. 6A). The binding of S100A16 to GRP78 was suppressed by treatment with BAPTA-AM (Fig. 6A). Quantitation of the western blot data for interaction between S100A16 and IRE1α with GRP78 is shown in Fig. 6B.

## Discussion

Fibrosis is the critical pathological feature of many chronic diseases including alcoholic liver cirrhosis, glomerulonephritis, viral myocarditis, respiratory distress syndrome [17]. In the kidney, renal interstitial fibrosis is characterized by the expansion of the space between the basement membrane of the renal tubules and the capillaries around the renal tubules through the secretion and deposition of matrix proteins [18]. Proteins that are destined to enter the secretory pathway first require proper folding for transportation, and this is one function of the ER. Protein folding and transport in the ER are facilitated by a variety of chaperone proteins [19]. Protein secretion is high in cells undergoing rapid proliferation, and a series of regular transcription and translation changes are activated to promote homeostasis. These changes are regulated by the UPR signaling pathway including PERK, ATF6, and IRE1α. The activation of UPR signals can promote new protein synthesis, improve protein folding, and induce the ER-associated degradation of misfolded cellular proteins [20, 21].

ER chaperones, such as ORP 150, GRP170, ERDJ4, GRP94, GRP78, Calnexin, and calreticulin, play the non-negligible role for ER normal function. GRP78, also known as BiP or HSPA5, is involved in various intracellular processes acting as a sensor for ER stress, promoting protein folding and assembly, degrading misfolded proteins, and regulating calcium homeostasis [22]. Recent work has shown that TGF-β increased GRP78 expression in human and mouse lung fibroblasts, while GRP78 inhibition significantly reduced the biomarkers expression of fibrosis, which included collagen and α-SMA [23]. Our previous mass spectrometry analysis also identified an interaction between GRP78 and S100A16 for the first time, suggesting a link between both GRP78 and S100A16 expression and fibrosis [14]. In this study, we demonstrate that TGF-β1 treatment induces GRP78 expression in a dose-dependent manner in HK-2 cells. These findings imply that ER stress plays a pro-fibrogenic role.

Few studies have considered a possible biological function of S100A16 in ER stress. S100A16 is known to promote lipid synthesis in HepG2 cells through the ER stress pathway [24]. Emmanuel Sturchler et al. found that [Ca^2+^] dose-dependent regulation of intracellular dynamics of S100A16 in Glioblastoma cell lines, that is to say when the intracellular calcium concentration is relatively high, S100A16 induces nuclear export and cytoplasmic transport. In contrast, the low concentration of calcium ions induces S100A16 nuclear translocation and nucleolar accumulation [25]. Our previous work showed that S100A16 promotes renal interstitial fibrosis in UUO mice and that, during kidney injury, S100A16 interacts with myosin-9 to promote the cytoskeletal reorganization of renal tubular interstitial fibrosis [14]. Other studies indicate that S100A16 is a multi-functional small protein that could affect the pathophysiological processes in many diseases, such as breast cancer [26], pancreatic cancer [27], and gastric cancer [28]. In the present study, IF confocal microscopy showed that overexpression of exogenous S100A16 resulted in colocalization of S100A16 and GRP78 mainly in the cytoplasm, whereas treatment with BAPTA-AM promoted S100A16 and GRP78 colocalization mainly in the endoplasmic reticulum and around the nucleus. We concluded that increased S100A16 expression drives Ca^2+^ accumulation in the cytoplasm and promotes the occurrence of ER stress, thereby aggravating the development of renal tubulointerstitial fibrosis in HK-2 cells.

Renal cell apoptosis and renal fibrosis are known to be triggered by long-term severe ER stress, as inhibition of ER stress can delay the development of fibrosis [29]. Similarly, endoplasmic reticulum stress induces apoptosis of renal cells mainly through two signaling pathways in UUO kidney: (1) ER stress induces autophosphorylation of PERK, which activates downstream eIF2α phosphorylation, thereby inducing ATF4 expression to up-regulate CHOP transcription factor. The upregulation of CHOP expression further inhibits the coding of anti-apoptotic genes (such as Bcl-2); (2) When ER stress becomes irreversible, sensor IRE1α will undergo homo-oligomerization, and further activates caspase-12 and JNK-mediated apoptosis pathways. During renal fibrosis linked with ER stress, TGF-β acts as a major regulator in renal tubular epithelial cells through the PI3K/Akt, Notch signaling, and Wnt/β catenin pathways [2, 30] via transcriptional factors such as Zeb1, Snail, TWIST1 [31]. Recent studies have identified a new mechanistic connection between ER stress and kidney injury through the ER-associated protein reticulon 1 (RTN1) in animal models and in humans [9, 32, 33]. The findings presented here now suggest an additional relationship between the S100 calcium-binding proteins, ER stress, and chronic kidney disease.

In conclusion, we demonstrated that the interaction between S100A16 and GRP78 interferes with ER stress signaling via IRE1α/XBP1 and promotes the occurrence and development of renal tubular fibrosis. As illustrated in Fig. 6C, under normal conditions, S100A16 is expressed only at low levels in HK-2 cells. A small portion of the S100A16 complexes with GRP78 and colocalizes on the ER. Simulation of renal tubular epithelial cells by damage factors such as TGF-β1 promotes an enhanced expression of S100A16 in the cells and its accumulation in the cytoplasm and cell membrane. GRP78 then leaves the ER and binds to S100A16 in the cytoplasm and cell membrane. ER stress promotes the release of IRE1α and the autophosphorylation of IRE1α activates the IRE1α pathway, which subsequently induces unconventional splicing of XBP1 mRNA. The end result is an ER stress-linked aggravation of tubular interstitial fibrosis. However, the mechanism by which ER stress regulates S100A16 expression needs further research and exploration.

## Materials and methods

### Reagents, plasmid constructs, and antibodies

Antibodies against S100A16, GRP78, IRE1α, XPB1, Fibronectin, and α-SMA for western blot analysis, GRP78 antibody for IHC, and Co-IP studies. The antibodies against β-actin and GAPDH were purchased from Proteintech (Chicago, IL, USA). The S100A16 antibody used for IHC, IF, and Co-IP studies were purchased from Sigma-Aldrich (St. Louis, Missouri, USA). The S100A16 and GRP78 antibodies for IF staining were purchased from Abcam (Cambridge, MA, USA). Protein A-agarose beads were purchased from EMD Millipore Corp (St. Louis, MO, USA). The antibodies of ubiquitin, p-IRE1α, EIF2α, p-EIF2α, p-PERK, PERK ATF6, and CHOP were purchased from Cell Signaling Technology (Billerica, MA, USA). The mouse IgG for Co-IP and secondary antibodies for western blotting and ER Tracker Blue-White were purchased from Thermo Fisher Scientific (Waltham, UK). Masson trichrome staining reagent was purchased from Solarbio (Beijing, China). Recombinant human TGF-β1 was purchased from PEPROTECH (Rocky Hill, NJ, USA). BAPTA-AM was purchased from MCE (New Jersey, USA). S100A16 overexpression lentivirus and negative control virus were purchased from Gene (Shanghai, China). Puromycin dihydrochloride and liposomal transfection reagent were purchased from YEASEN (Shanghai, China).

### Mice and animal models

Male C57BL/6 J (WT) mice and S100A16 transgenic (S100A16^Tg^) mice (*n* = 16 in each group) (8 weeks, 20–24 g) were obtained from the Animal Center of Nanjing Medical University. All animal procedures are conducted in accordance with experimental animal ethics, and the animals are raised in accordance with the guidelines of the Animal Committee of Nanjing Medical University. S100A16^Tg^ mice were generated as previously described [34]. The UUO procedure was conducted by left ureteral ligation with 6-gauge silk sutures in mice at 12 weeks of age. The UUO and contralateral kidneys were removed on day 7 after UUO. Part of each kidney is fixed with 10% formalin for histologic and IHC staining. Another portion of each kidney was stored at −80 °C for extraction of RNA and protein.

### Cell culture and TGF-β1, BAPTA-AM treatment

Human kidney-2 (HK-2) cells, which are human renal proximal tubular epithelial cells, were cultured in DMEM-F12 medium (Gibco America) with 10 % fetal bovine serum and incubated at 37 °C in an atmosphere of 5% CO_2_. After reaching a density of 50–60%, the cells were growth-arrested in a serum-free medium for 24 h and then treated with 0, 10, 20, or 30 nM of TGF-β1 and 0 or 10 µM BAPTA-AM for 24 h.

### Establishing the HK-2 cell line with stable S100A16 overexpression

Lentiviral particles containing full-length human S100A16 cDNA were cloned into pcDNA3.1 using the EcoRI restriction site. To generate stable lines of S100A16-overexpressing cells, the S100A16 lentivirus was mixed with transfection reagent and added to the HK-2 cells. The cells were transfected for 48 h and then cultured in a puromycin-containing culture medium for 14 days to obtain the HK-2 cell line with stable S100A16 overexpression.

### Western blotting and co-IP assay

Total cell protein lysates from mice or HK-2 cells were resolved by 8 or 12% Sodium dodecyl-sulfate polyacrylamide gel electrophoresis on gels and electroblotted onto nitrocellulose membranes. The blotted membranes were incubated overnight at 4 °C with primary antibodies at various dilutions after the membranes were blocked with 5% skimmed milk in Tris-buffered saline with 0.1% Tween-20 for 3 h at room temperature, and then the horseradish peroxidase-conjugated secondary antibodies were incubated. The protein bands were detected by enhanced chemiluminescence solution (WesternBright^TM^ ECL), and images were obtained using the Tanon exposure meter and the Image Quant ECL system (PerkinElmer Life Sciences, Wellesley, MA). Western blotting data were obtained using the Image J software.

For IP studies, cell lysates were first collected from wild-type or S100A16-overexpressing HK-2 cells by lysing with NP40 lysis buffer. The protein concentrations of the lysates were determined by bicinchoninic acid assays, and then 1 mg protein was incubated with anti-S100A16 and anti-GRP78 overnight at 4 °C. The antibody-protein mixture was then incubated with 100 μL protein A beads for 2–6 h. The resulting immunocomplexes were washed three times in NP40 lysis buffer, degenerated with SDS loading buffer, and then incubated with anti-S100A16, anti-GRP78, and anti-IRE1α.

### IF staining

For IF staining, HK-2 cells were fixed in 4% paraformaldehyde for 4 h at room temperature and permeabilized for 10 min with phosphate-buffered saline (PBS) containing 0.2% Triton (Sigma). In all, 5% BSA in PBS was added for 30 min to block for nonspecific binding in HK-2 cells, then cells were incubated overnight with primary antibody against S100A16 and GRP78. The cells were washed with cold PBS, followed by incubation with fluorescein isothiocyanate (FITC)-conjugated secondary antibody for 90 min. All images were obtained using an Olympus confocal microscope.

### ER tracker

In brief, HK-2 cells were incubated with 5 µM ER probe (ER Tracker Blue-White DPX) at 37 °C and 5% CO_2_ for 30 min, and then washed twice with Hanks’ Balanced Salt Solution, fixed with 4% paraformaldehyde for 30 min. The confocal microscope (Carl Zeiss LSM880) was used to take photos.

### RNA extraction, purification, and real-time PCR analyses

Total RNA was extracted from HK-2 cells using TRIzol reagent according to the manufacturer’s instructions (Thermo, USA). RNA was then reverse transcribed to cDNA with a ReverTra Ace qPCR RT kit (TOYOBO, China). The mRNA levels were determined using SYBR Green Master Mix (Applied Biosystems) and the Applied Biosystems StepOne Plus Real-Time PCR system with housekeeping gene GAPDH used as an internal control. All reactions were conducted using the following cycling parameters: 95 °C for 10 min, followed by 35 cycles of 94 °C for 15 s and 60 °C for 50 s. All fold changes in expression relative to the control group were calculated using the 2^−ΔΔCt^ method. The following primer sequences were used: human S100A16: forward 5′-TTGGATCCGGAGATGTCAGACTGCTACAC-3′ and reverse 5′-TTACGCGTAAAGGGGTCTCTAGCTGCTG-3′; human GAPDH: forward 5′-ATGGGGAAGGTGAAGGTCG-3′ and reverse 5′-GGGGTCATTGATGGCAACAATA-3′; human GRP78: forward 5′-TGACCAGAATCGCCTGACAC-3′ and reverse 5′-TGTCAGCATCTTGGTGGCTT-3′; human β-Actin: forward 5′-AGGATTCCTATGTGGGCGAC-3′ and reverse 5′-ATAGCACAGCCTGGATAGCAA-3′.

### RT-PCR assay for XBP1 splicing detection

Platinum® Taq DNA Polymerase High Fidelity (Invitrogen) was used for PCR with using primers flanking the splice site. The primer sequences for XBP1s detection were: XBP1: 5′-TTACGAGAGAAAACTCATGGCC-3′ (sense) and 5′-GGGTCCAAGTTGTCCAGAATGC-3′ (antisense); β-Actin: 5′-AGGATTCCTATGTGGGCGAC-3′ and reverse 5′-ATAGCACAGCCTGGATAGCAA-3’. The PCR conditions were 95 °C for 2 min, followed by 95 °C for 30 s, 59 °C for 30 s, and 66 °C for 30 s for 31 cycles. The length of XBP1 and XBP1s was 288 bp and 257 bp, respectively. Two PCR products were obtained by 3% agarose gel electrophoresis and stained with Gel-Red.

### Immunohistochemistry

Mouse kidney samples were fixed, embedded in paraffin, and sectioned at 3 or 4 μm thickness for IHC. IHC staining was performed with routine protocols using rabbit polyclonal anti-S100A16 and anti-GRP78 antibodies. The primary antibody was incubated at 4 °C overnight, and washed three times with PBS for 5 min. Finally, a horseradish peroxidase-conjugated secondary antibody is applied.

### Masson trichrome staining

The sections were routinely deparaffinized, stained with Weigert iron hematoxylin staining solution for 5–10 min, differentiated for 5–15 s in acid ethanol differentiation solution, and washed with water. Masson blue solution was then applied for 3–5 min. The sections were washed with water, stained with Ponceau red magenta solution for 5–10 min, and washed with a phosphomolybdic acid solution for 1–2 min. The sections were then directly dyed with aniline blue staining solution, washed with a prepared working solution for 1 min, then with 95% ethanol, dehydrated with anhydrous ethanol three times, and then treated with xylene three times until transparent for observation.

### Statistical analysis

All of the experimental results were statistically analyzed using GraphPad Prism 8.0 software (San Diego, CA, USA). Data were expressed as the mean ± SE. We used the *t* test and analysis of variance to determine statistical differences between groups. *P* values <0.05 were considered significant.

## Supplementary information

Supplemental data

## Data Availability

All data generated during this study are included in this published article and its supplementary information files.
