# Supplementary material for: The interaction of S100A16 and GRP78 actives endoplasmic reticulum stress-mediated through the IRE1α/XBP1 pathway in renal tubulointerstitial fibrosis
Source: Cell Death Dis. 2021 Oct 13;12(10):942. doi: 10.1038/s41419-021-04249-8 (PMC8514525; doi:10.1038/s41419-021-04249-8)

Supplementary Data

Title: The interaction of S100A16 and GRP78 actives endoplasmic reticulum stress mediated through the IRE1α/XBP1 pathway in renal tubulointerstitial fibrosis

Authors: Runbing Jin[1#], Anran Zhao[1#], Shuying Han[1], Dan Zhang[1], Hui Sun[1], Min Li[2], Dongming Su[2] and Xiubin Liang[1, 3, *]

Figure S1. **a.** The mRNA levels of S100A16 and GRP78 determined by real-time PCR in HK-2 cells treated with TGF-β1. **b.** The expressions of S100A16 and GRP78 were higher in S100A16^{Tg} kidneys than in wildtype kidneys. Immunohistochemical staining of S100A16 and GRP78 in the sham, S100A16^{Tg} (sham), kidney-obstructed UUO, and S100A16^{Tg} (kidney-obstructed UUO) mice. Scale bar =100 μm. **c.** Overexpression of S100A16 increased the protein expression levels of p-PERK/PERK, ATF6, and CHOP.

Figure S1

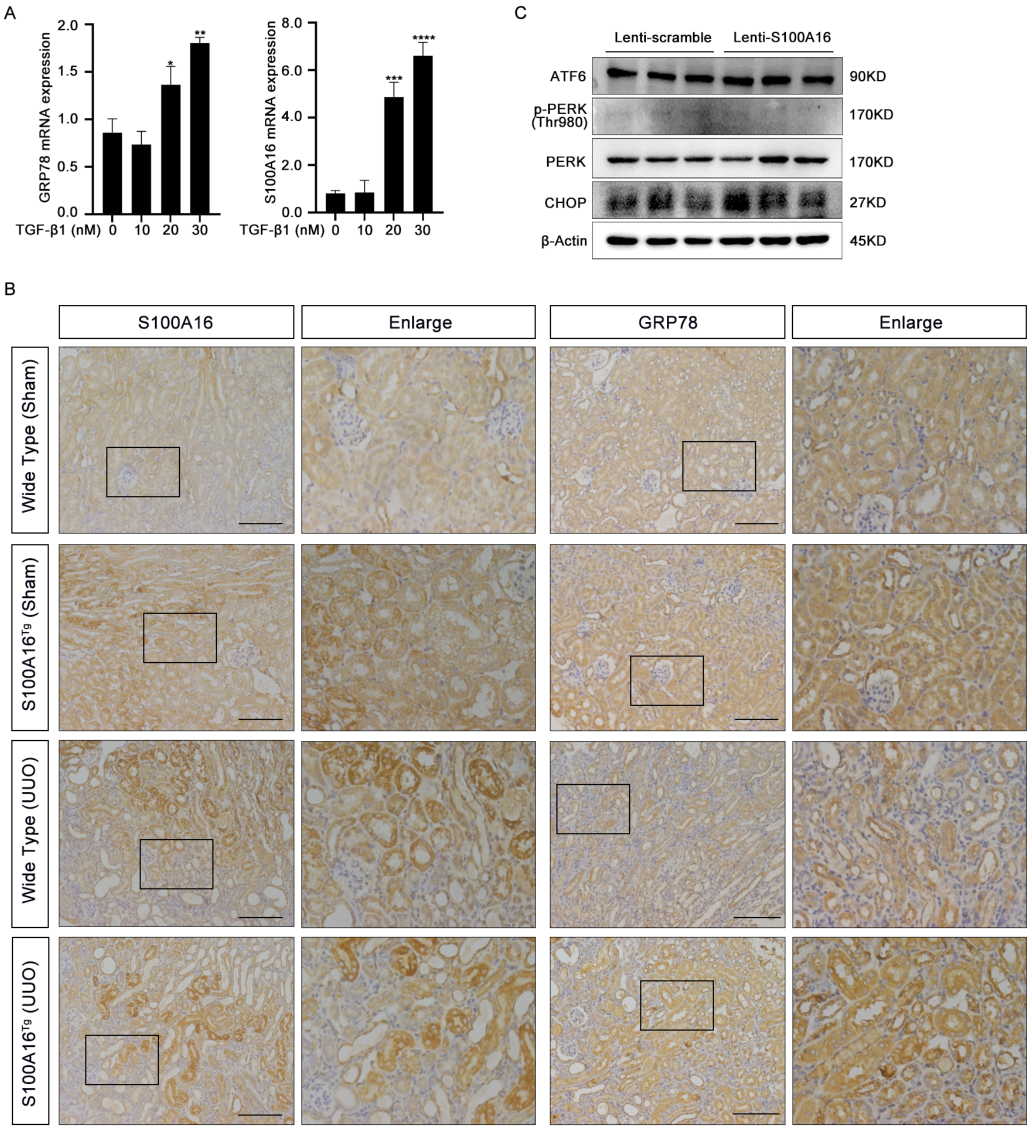

Figure S2. **a.** Intracellular calcium concentrations determined with fluorescent the Ca^{2+} indicator probe Rhod-2 AM in normal and S100A16 overexpressing HK-2 cells treated with BAPTA-AM (10 μM). *P < 0.05, **P < 0.01, ***P < 0.001. **b.** The mRNA levels of S100A16 and GRP78 determined by real-time PCR after BAPTA-AM treatment of Lenti-scramble and S100A16 overexpressing HK-2 cells.

Figure S2

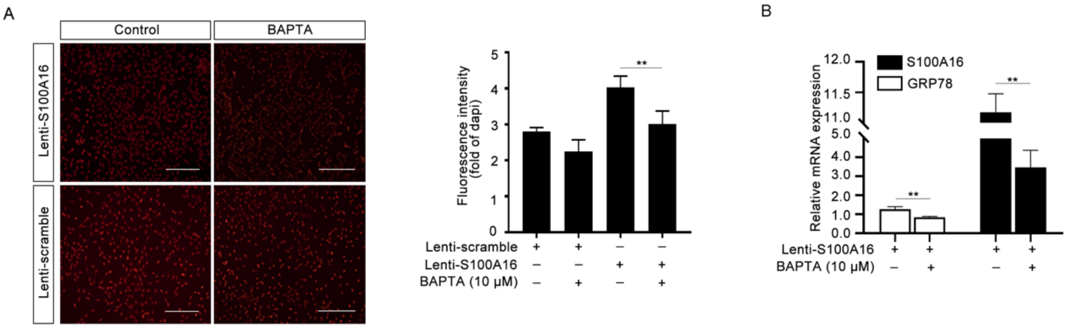

Supplement: Supplementary file 1 — Supplemental data [file 41419_2021_4249_MOESM1_ESM.pdf]
